# Against a normative view of folk psychology

**DOI:** 10.3389/fpsyg.2014.00598

**Published:** 2014-06-16

**Authors:** Meredith R. Wilkinson

**Affiliations:** Division of Psychology, Faculty of Health and Life Sciences, School of Applied Social Sciences, De Montfort UniversityLeicester, UK

**Keywords:** folk psychology, normative, reasoning, descriptive, theory theory, simulation theory

Recently Elqayam and Evans (2011) have proposed that researchers studying human thinking should be moving away from normative accounts that specify how we “ought” to reason to a more descriptivist framework that describes *how* we reason. This is an approach that I very much support. The aim of the present article is to demonstrate how this can be applied to the study of mental state reasoning in terms of folk psychology (FP). Folk psychology refers to our everyday ability to attribute mental states to other people, including their beliefs, desires, intentions and so forth (e.g., Ratcliffe and Hutto, 2007). I do not want to deny that FP *can* be normative. Indeed, there are many instances where normative responding is required. For example, in the traditional false belief task (e.g., Baron-Cohen et al., 1985) there is a “right” or “wrong” answer - a single norm paradigm (Elqayam and Evans, 2011). However, it may be the case that FP is normative in certain circumstances (e.g., the false belief task) but as I shall suggest below this is not always the case. By viewing FP as normative what researchers end up doing is ignoring the *processes* of how such inferences arise. What I want to propose is that viewing FP as normative is problematic since it reduces mental state inferences to simply being “right” or “wrong.” I propose that moving away from a normative agenda in FP and embracing a more descriptivist framework proves extremely useful for our understanding of how we understand others' minds.

## Folk psychology as a normative framework

One factor that philosophers have proposed regarding FP is that it is normative in nature. One of the earliest claims of this was made by Dennett (1989) who argued:

“Folk psychology, then, is idealized in that it produces its predictions and explanations by calculating in a normative system; it predicts what we will believe, desire, and do, by determining what we ought to believe, desire, and do” (Dennett, 1989 p. 52).

Dennett (1989) views FP as a form of mindreading but has the perspective of the intentional stance. His perspective appears to be normative in nature specifying both a normative framework and an ought stance. This sense of normativism in FP extends to recent literature:

“Even on the standard view, then, folk psychology is not just an explanatory/predictive practice, it is also, in a sense, a normative practice: a practice of showing how people's performances lives up to certain norms and thereby become, in that special way, intelligible. Although folk psychologists may have some context-specific views about what others will do—based, for instance, on experience—the bulk of these views will be heavily influenced by norm-governed judgments about what others ought to do, what it makes sense to do in the circumstances” (McGeer, 2007, p. 141).

The above quotation appears to be arguing that we can in our FP responses generate normative responses. Just as we have a normative rule that when driving you should stop at a red light, what is being implied here is that we have a sense of what people “ought” to do in certain situations. Viewing FP in a normative framework is a view that exists till the present day:

“Whatever focus one adopts, judgments of the rational or scientific status of elements in folk psychology are inevitably normative judgments, based on comparisons between what ordinary folk do with some prescriptive scientific account” (Fletcher, 1995, pp. 43–44).

Such a perspective sees FP as confirming to rationality and having an analogy with science. However, if FP is to have an analogy with science then we may to some degree want to subject it to empirical testing. However, Churchland (1991) argues that empirical testing may do little for FP:

“Folk psychology, insist others is radically unlike the examples cited. It does not consist of laws. It does not support causal explanations. It does not evolve over time. Its central purpose is normative rather than descriptive. And thus, it is not the sort of framework that might be shown to be radically defective by sheerly empirical findings” (Churchland, 1991, p. 51).

I think that this quote is somewhat problematic as FP does support causal explanations and it has evolved over time. For example, the use of neuroscience to examine FP (e.g., Ruby and Decety, 2001). I hope to have demonstrated in this section how multiple theorists view FP as normative and now aim to demonstrate what is problematic about doing so.

## What is problematic about viewing folk psychology as normative?

Whilst I accept that there is normative responding in FP, for example, it is normative to assume that if we push someone off a seat on the bus so that we can sit down then they will be angry I believe that viewing FP as normative is problematic since it reduces mental state reasoning to “right” or “wrong” answers and indeed a “right” way to reason (as indicated by the quotations above). Admittedly, FP is a single norm paradigm (Elqayam and Evans, 2011) so does not face the difficulty that reasoning research does of having multiple normative accounts to arbitrate between.

I agree with Elqayam and Evans (2011) when they claim that normativism has biased the study of thinking and feel that this can be applied to the study of FP. FP has repeatedly made use of tasks of false belief in order to examine mentalizing. I believe that this is problematic since it has led to a very restricted range of tasks being studied. If we were to move away from a normative perspective of FP then this would open many more doors to examine mentalizing since far too much attention, from my perspective, has been focused on the false belief paradigm. Thus, what is happening here is that people are either attributed with having a capacity to engage in FP reasoning or not. I believe that this is problematic since there is much more to FP then the false belief task and much more to the false belief task than FP understanding (Bloom and German, 2000). I demonstrate within the next section how a descriptivist study of FP may work.

A final problem with viewing FP as normative is that although there are clear cut cases, as in the false belief task, where there is a “right” and “wrong” answer in tasks of mental state reasoning this isn't always going to be the case. I believe that if something is to be *fully* normative then it should always be the case that there is a right and wrong response. For example, if we are informed by our friend that her boyfriend has ended their relationship we may assume that she will be devastated. However, other factors may influence that judgment such as if she wanted to separate with him then you may believe that she will be relieved. However, it is still possible that she will be upset as he separated with her first. What I aim to demonstrate here is that there is not always a clear cut answer with FP and therefore we should embrace a more descriptive framework rather than a normative one.

## What are the advantages of a descriptivist account?

I have argued that viewing FP as normative can be severely problematic. I am not the only theorist who takes this view. Andrews (2012) argues that “the study of folk psychology is a descriptive endeavor, as opposed to a normative one.” (Andrews, 2012, p. 251). Thus, what we should be aiming to do as researchers is not provide an account of how people *ought* to reason but provide an account of how they do reason using appropriate theories and experimental methodologies.

A recent descriptivist approach to FP comes from Wilkinson and Ball (2013) who provide a dual-process perspective to theorizing and simulation. I draw the link to the theorizing vs. simulation theory debate here since it is the ideal type of question that those who study FP should be asking in terms of the cognitive processes which underlie FP, I am not saying that the term FP necessarily refers to the theorizing vs. simulation debate itself. Theorizing refers to understanding mental states via the adoption of theories which link mental states, behavior and the environment together (e.g., Carruthers, 1996). Whereas simulation proposes that we reason about others' mental states via controlled processes of simulation either from a third-person (e.g., Goldman, 2006) or first-person (e.g., Gordon, 1986) perspective. According to Wilkinson and Ball theorizing is viewed as synonymous with intuitive reasoning and simulation is viewed as synonymous with reflective reasoning within a dual-process framework (e.g., Evans, 2010). As such, theorizing can be viewed as possessing the characteristics of being fast, automatic, low effort, high capacity and independent of working memory resources whereas simulation is slow, controlled, high effort, low capacity and dependent upon working memory resources.

According to Wilkinson and Ball (2013) people can either choose to theorize or simulate. It is possible for them to engage in both with people skipping between theorizing and simulation and vice versa. This reflects the hybrid nature of theorizing and simulation (see also Mitchell et al., 2009). Wilkinson and Ball note that conflict may arise between the responses generated by theorizing and simulation and this can be overcome with a conflict resolution mechanism which is analogous to Evans' (2009) type 3 reasoning. Conflict does not have to arise though and one response may just be generated. The advantage of viewing FP in this manner is that it promotes a program of research which focuses on the question of ‘how’ people reason and not just how they ought to reason. It further avoids the tricky issue of rationality, something which in mental state reasoning would be very difficult to examine since what is rational for one agent is not necessarily rational for another. I believe that this addresses the issue raised above regarding how viewing FP as normative has led to a bias in how it is studied. Wilkinson et al. (2010) used think aloud protocols where they asked participants to think aloud whilst working through regret-orientated counterfactual scenarios and then coding participants' verbalizations for instances of theorizing and simulation. Adopting this method enabled Wilkinson et al. (2010). To gain a measure of *how* people reason which gives a much richer insight than whether someone answered correctly or incorrectly.

Wilkinson and Ball (2013) are not the only researchers to link FP to dual-process theories. Bohl and van den Bos (2012) propose that theory of mind requires reflective reasoning whereas interactionism requires intuitive reasoning. Whilst their account differs from Wilkinson and Ball since they propose that theory of mind consists of both intuitive and reflective processes both demonstrate a move toward viewing mental state reasoning in dual-process terms (see also Apperly and Butterfill, 2009).

I, like Andrews (2012), believe that FP is descriptive rather than normative in nature and Andrews aims to develop an account which is more descriptively accurate than normative. It is only via viewing FP as descriptive can real progress be made into examining the complexities of our abilities to engage in mental state reasoning of both ourselves and other people. Within the reasoning literature some authors endorse a “soft normativsm” perspective (e.g., Stupple and Ball, 2011) whereby they propose that normative constructs can feed into a descriptivist framework. To some degree I endorse this perspective since there are normative rules which govern how we expect others to feel. However, I do believe that the descriptivist framework of Wilkinson and Ball (2013) enables a much richer account of the cognitive processes in mental state reasoning than any normative only perspective permits.

### Conflict of interest statement

The author declares that the research was conducted in the absence of any commercial or financial relationships that could be construed as a potential conflict of interest.
